# The Role of Glucocorticoid Receptors in Dexamethasone-Induced Apoptosis of Neuroprogenitor Cells in the Hippocampus of Rat Pups

**DOI:** 10.1155/2013/628094

**Published:** 2013-01-14

**Authors:** Chun-I Sze, Yung-Chieh Lin, Yuh-Jyh Lin, Ting-Hui Hsieh, Yu Min Kuo, Chyi-Her Lin

**Affiliations:** ^1^Department of Cell Biology and Anatomy, College of Medicine, National Cheng Kung University, Tainan, Taiwan; ^2^Department of Pediatrics, College of Medicine, National Cheng Kung University, Tainan, Taiwan

## Abstract

*Background*. Dexamethasone (Dex) has been used to reduce inflammation in preterm infants with assistive ventilation and to prevent chronic lung diseases. However, Dex treatment results in adverse effects on the brain. Since the hippocampus contains a high density of glucocorticoid receptors (GCRs), we hypothesized that Dex affects neurogenesis in the hippocampus through inflammatory mediators. *Methods*. Albino Wistar rat pups first received a single dose of Dex (0.5 mg/kg) on postnatal day 1 (P1) and were sacrificed on P2, P3, P5, and P7. One group of Dex-treated pups (Dex-treated D1D2) was given mifepristone (RU486, a GCR antagonist) on P1 and sacrificed on P2. Hippocampi were isolated for western blot analysis, TUNEL, cleaved-caspase 3 staining for cell counts, and morphological assessment. Control pups received normal saline (NS). *Results*. Dex reduced the developmental gain in body weight, but had no effect on brain weight. In the Dex-treated D1D2 group, apoptotic cells increased in number based on TUNEL and cleaved-caspase 3 staining. Most of the apoptotic cells expressed the neural progenitor cell marker nestin. Dex-induced apoptosis in P1 pups was markedly reduced (60%) by pretreatment with RU486, indicating the involvement of GCRs. *Conclusion*. Early administration of Dex results in apoptosis of neural progenitor cells in the hippocampus and this is mediated through GCRs.

## 1. Introduction

Corticosteroids are used in preterm infants to suppress inflammation, to facilitate extubation, and/or to prevent chronic lung diseases [1–3]. However, such early dexamethasone (Dex) therapy can result in an adverse neurodevelopmental outcome [4, 5]. For example, Dex treatment reduces cerebral gray matter volume without affecting white matter and the basal ganglia, suggesting that Dex affects only certain cells in the brain [6–8].

Neurons in the dentate gyrus (DG) of the hippocampus continue to divide after term and therefore remain vulnerable to the adverse effects of steroids during the early postnatal period [9–11]. Cells in the hippocampus have a high density of glucocorticoid receptors (GCRs) [12] suggesting that they could be affected by Dex [8–13]. Dex is known to change synaptic plasticity in the hippocampus [14]. As rat and human develop over different embryonic time scales [15], rat pups on P1 correspond to the human fetus at about week 22 to 24 of gestation. The equivalence in development is reflected in brain weight, neurochemistry, electroencephalography, and synaptogenesis [16]. Therefore, rat pups can be used as an animal model for human preterm infants undergoing drug exposure [17].

Here, we studied the effects of single-dose Dex therapy and the role of GCRs on hippocampal development.

## 2. Materials and Methods

### 2.1. Animals

The study was approved by the Animal Care and Use Committee of National Cheng Kung University. Time-dated pregnant Albino Wistar rats (body weight 250–300 g) were used. Food and water were provided *ad libitum*. The dams were allowed to deliver naturally on gestational day 21 ± 1. Animals were kept in a ventilated room at 22 ± 2°C under a 12/12 h light/dark cycle. The day of birth was designated P0.

### 2.2. Study Design

In a preliminary study, each litter was divided into two groups: the Dex group received a dose of Dex phosphate (0.1, 0.2, or 0.5 mg/kg, i.p.) (Oradexon, 4 mg/mL, Organon, the Netherlands) and controls received an equal volume of normal saline (NS). Compared with the controls, changes in apoptosis were only observed with Dex 0.5 mg/kg, so this dose was used in the subsequent experiments. On day 1 (P1) pups received a single dose of 0.5 mg/kg Dex or NS. On P2, P3, P5, and P7, pups were anesthetized and euthanized with 10% chloral hydrate (W/V, 300 mg/kg, Riedel-de Haen, Germany) and then infused transcardially with NS. The body and isolated brain weights were measured to the nearest milligram. Brain tissues were processed histologically following standard protocols and cut serially at 5 *μ*m in the coronal plane.

### 2.3. Immunohistochemical and Immunofluorescence Staining

Brain sections were matched to the E22 coronal plates 12 to 15 of the prenatal rat brain development atlas [18]. Sections were stained by immunohistochemical (IHC) and/or immunofluorescence (IF), TUNEL, and double-IF methods, using the protocols recommended by the manufacturers or described previously [19]. The following TUNEL detection kits and primary antibodies were used: TdT-Frag EL DNA fragmentation detection kit, ApopTag Red *in situ* apoptosis detection kit (Calbiochem, San Diego, CA), antibrain-derived neurotrophic factor (diluted 1 : 2000), and rabbit antiglucocorticoid (diluted 1 : 50) (Santa Cruz Biotechnology, Santa Cruz, CA), mouse antinestin (diluted 1 : 200; Santa Cruz Biotechnology), anti-OX-6 (diluted 1 : 50; AbD Serotec, Oxford, UK), and anti-NeuN (diluted 1 : 200; Chemi-Con, Temecula, CA), mouse antiactin and anti-pCNA (diluted 1 : 10000 and 1 : 1000, resp.; Millipore, Billerica, MA), and cleaved caspase 3 (diluted 1 : 100; Cell Signaling, Boston, MA). All sections were matched to the same anatomical sites for comparing cell counts between the Dex-treated and control groups. For IF staining, biotinylated anti-rabbit and anti-mouse IgG (1 : 400; Victor, Burlingame, CA) were used as the secondary antibodies; for IHC staining goat anti-rabbit IgG, H&L Chain specific Texas Red conjugate and rat anti-mouse IgG H&L Chain specific fluorescein conjugate (diluted 1 : 400; Calbiochem, San Diego, CA) were used as secondary antibodies. Sections were also double stained using the above primary antibodies and dilutions.

### 2.4. TUNEL Assay

The sections were subjected to TUNEL (Oncogene Research, Cambridge, MA) and IF staining. The signal was detected by the streptavidin-horseradish peroxidase conjugate and diaminobenzidine reaction.

### 2.5. Counts of Cells Stained for TUNEL, Cleaved-Caspase 3, and NeuN

Sections were examined by light microscopy and the images captured by a video camera coupled to a desktop computer (Eclipse 80i, Nikon, Japan). TUNEL-positive cells were identified and counted at 400x magnification. The numbers of TUNEL-positive cells in the DG, cornu ammonias 1 (CA1), CA2, and CA3 in the hippocampus were counted. For comparison with the control, sections at the same level were analyzed. Four sections were counted for each pup and the results were averaged. Cleaved-caspase 3- and NeuN-positive cells were counted at 400x magnification in four, 1 mm^2^ areas in the DG. Data were validated by TissueGnostics FACS-like Tissue Cytometry software (Vienna, Austria). This method was also applied to IHC, IF, and double-stained cells in subsequent experiments.

### 2.6. Tissue Dissection and Western Blot Analysis

The hippocampi were dissected from the pups on P2, one day after Dex or NS treatment (D1D2). Western blots were performed on cytosolic and nuclear fractions of the hippocampus homogenates as described previously [19]. Briefly, 20 *μ*g protein homogenate, determined by the Bradford protein assay (Bio-Rad, Hercules, CA), was separated by SDS-PAGE, blotted onto nitrocellulose (Hy-Bond, Amersham, Arlington Heights, IL) and blocked with nonfat dry milk. Blots were incubated with specific primary antibody, followed by incubation with horseradish-peroxidase-conjugated secondary antibodies (Abcam, Cambridge, UK) and detected by enhanced chemiluminescence (Bio-Rad). Samples were normalized to actin and proliferating cell nuclear antigen (pCNA**)** proteins (EMD Millipore). Each experiment was repeated at least three times.

### 2.7. Mifepristone (RU486) Treatment

The GCR antagonist, RU486 (Tocris Bioscience, Ellisville, MO), was dissolved in 100 mM DMSO. Thirty minutes prior to Dex injection, P1 pups received 25 mg/kg RU486 or vehicle intraperitoneally [20]. The RU486- and DMSO-treated pups (D1D2) with or without Dex treatment were euthanized on P2, and morphological and biochemical analyses were carried out as described above.

### 2.8. Statistical Analysis

Quantitative results are expressed as mean ± standard error (SE). Statistical analyses were performed using one- or two-way ANOVA with a multiple comparisons posttest or the Wilcoxon signed-rank test as appropriate. *P* values < 0.05 were considered statistically significant. The body and brain weight results were analyzed by mixed-model ANOVA with age as the within-subject factor and Dex as the between-subject factor.

## 3. Results

### 3.1. Body and Brain Weights

Pups that had received Dex (0.5 mg/kg) showed reduced body weights. In contrast, no difference in the developmental growth of brain weight was found between the Dex and control groups (Table 1).

### 3.2. Apoptotic Cell Death

**Figure 1:**
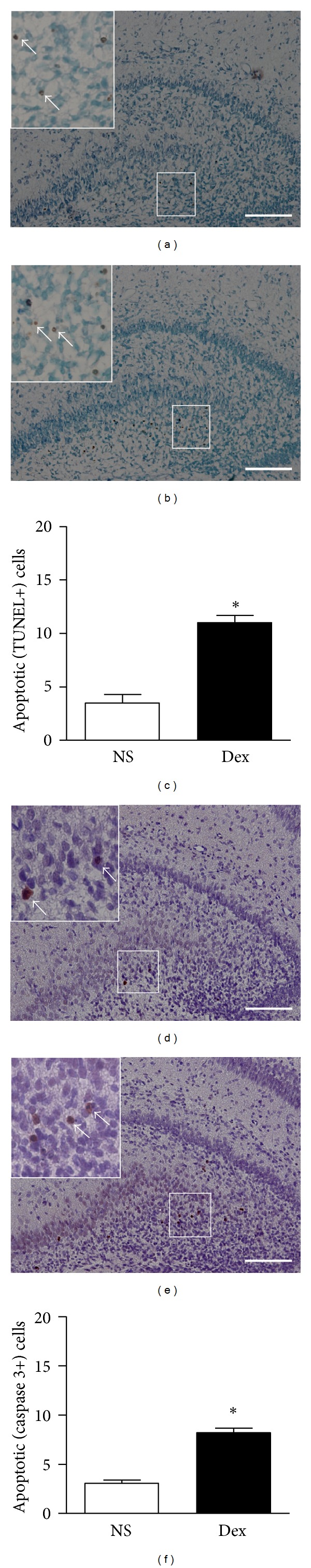
TUNEL staining of dentate gyrus (DG) P1 D1D2 pups treated with dexamethasone (Dex) or normal saline (NS) shows apoptotic cells stained brown (arrows). The Dex-treated D1D2 DG had more apoptotic cells (b) than NS control (a). High magnification photomicrographs of TUNEL-positive cells are shown in the upper left corner. TUNEL-positive cell counts revealed that the number of apoptotic cells in the Dex-treated D1D2 DG was increased when compared to that of NS control pups (*n* = 6, *P < 0.05) (c). Cleaved-caspase 3 staining of the DG from P1 D1D2 pups with or without Dex treatment showed apoptotic cells (arrows) ((d) and (e)). High magnification photomicrographs of cleaved-caspase 3-positive cells are shown in the upper left corner. The numbers of apoptotic cells in Dex-treated pups were increased compared to those of NS control (f) (*n* = 6, *P < 0.05). Magnification, 200x; scale bars, 100 *μ*m; inset magnification, 400x. P1 D1D2: P1 pups receiving treatment on postnatal day 1 and sacrificed on day 2.

More TUNEL-positive and cleaved-caspase 3-positive cells were found in the DG of P1 D1D2 Dex-treated group (Figures 1(b) and 1(e)), with an increase to 2.7- to 3-fold (Figures 1(c) and 1(f)) with respect to the control (Figures 1(a) and 1(d)).

### 3.3. Cell Counts and Coexpression of Apoptosis and Neuronal Maturation Markers

**Figure 2:**
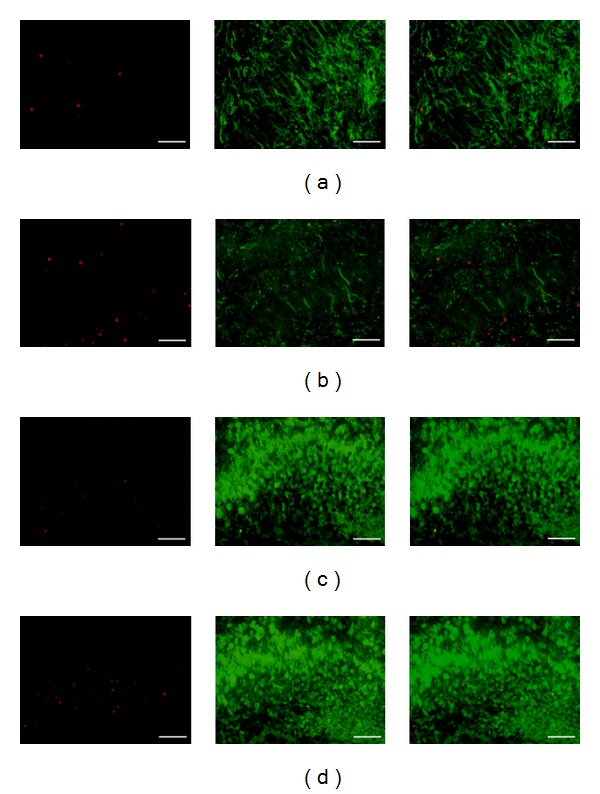
TUNEL, nestin, and NeuN expression in the hippocampus of D1D2 pups treated with normal saline (NS) or Dex. Representative double-IF micrographs demonstrate TUNEL (red) and nestin (green) staining ((a) and (b)), and TUNEL (red) and NeuN (green) staining ((c) and (d)). Cells coexpressing two proteins are merged and show as yellow or orange. Magnification, 400x, scale bars, 50 *μ*m. Dex-treated D1D2: P1 pups receiving dexamethasone on postnatal day 1 and sacrificed on day 2.

**Figure 3:**
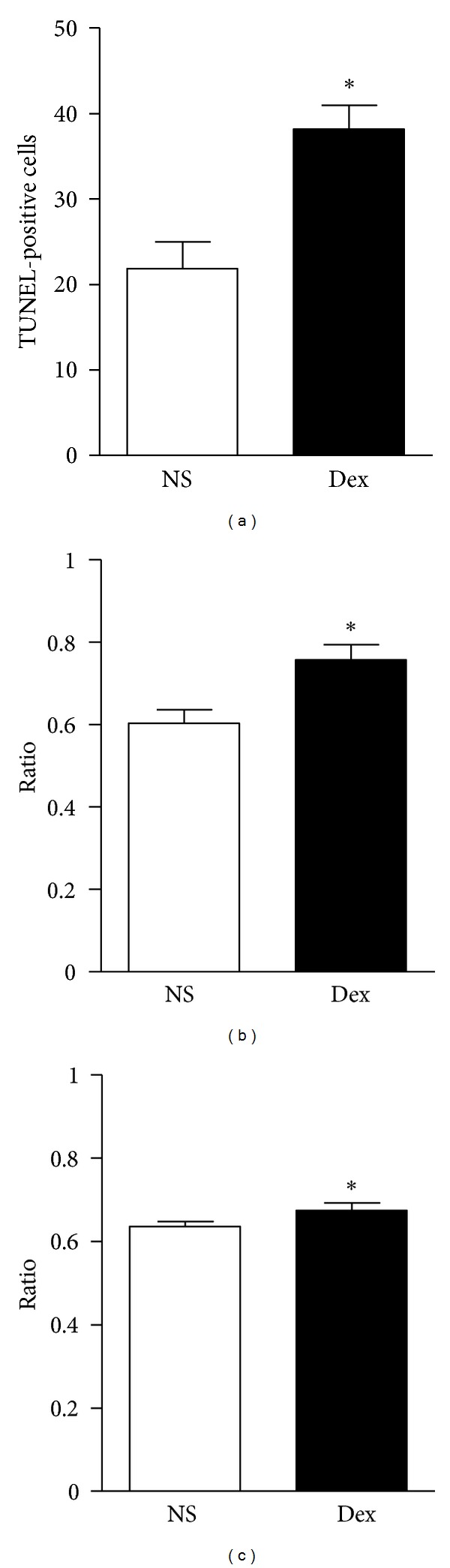
TUNEL-positive cells were more numerous in the hippocampus of Dex-treated D1D2 pups than in control (*n* = 6, *P < 0.05) (a) and the ratio of TUNEL-positive cells which coexpressed nestin to total TUNEL-positive cells was higher in the Dex treated than in the control groups (*n* = 6, *P < 0.05) (b). The ratio of TUNEL-positive cells coexpressing NeuN to total TUNEL-positive cells was higher than that of control (*n* = 6, *P < 0.05) (c). Dex-treated D1D2: P1 pups receiving dexamethasone on postnatal day 1 and sacrificed on day 2.

Representative results of TUNEL, nestin, and NeuN staining are shown in Figure 2. We found about twice as many TUNEL-positive cells in the Dex-treated D1D2 group (38.1 ± 1.1) compared with the control (21.8 ± 1.2; *P* < 0.05) (Figure 3(a)). The Dex-treated group had a greater proportion of TUNEL-positive cells (0.75 ± 0.01) that coexpressed nestin than control (0.61 ± 0.01) (Figure 3(b)). The proportion of TUNEL-positive cells coexpressing NeuN was also greater in the Dex-treated group (0.68 ± 0.10) than in control (0.64 ± 0.01; *P* < 0.05) (Figure 3(c)).

### 3.4. Glucocorticoid Receptors (GCRs) and Mifepristone (RU486) in Dex-Induced Apoptosis

**Figure 4:**
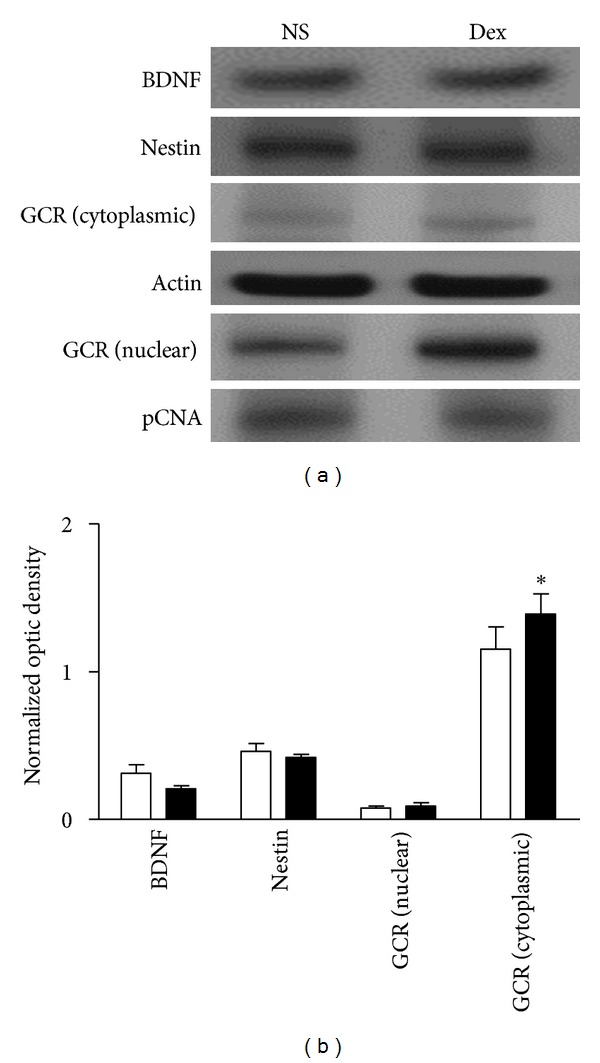
Dexamethasone (Dex) treatment increased levels of nuclear glucocorticoid receptors (GCRs), but not cytosolic GCRs, total brain-derived neurotrophic factor (BDNF) or total nestin in the hippocampus of P1 pups. (a) Representative western blots of hippocampal protein extracts obtained from P1 D1D2 pups, normal saline (NS, control) (left lane), and Dex (right lane). (b) Signals from BDNF, nestin, and cytosolic GCRs were normalized to actin and signals from nuclear GCRs were normalized to pCNA. Quantitative results showed no difference in BDNF, nestin, and cytosolic GCR expression between Dex-treated and control pups. The nuclear fraction of GCR expression in Dex-treated pups differed from control (*n* = 6, *P < 0.05). P1 D1D2: P1 pups receiving treatment on postnatal day 1 and sacrificed on day 2; white and black bars represent control and Dex-treated pups, respectively.

**Figure 5:**
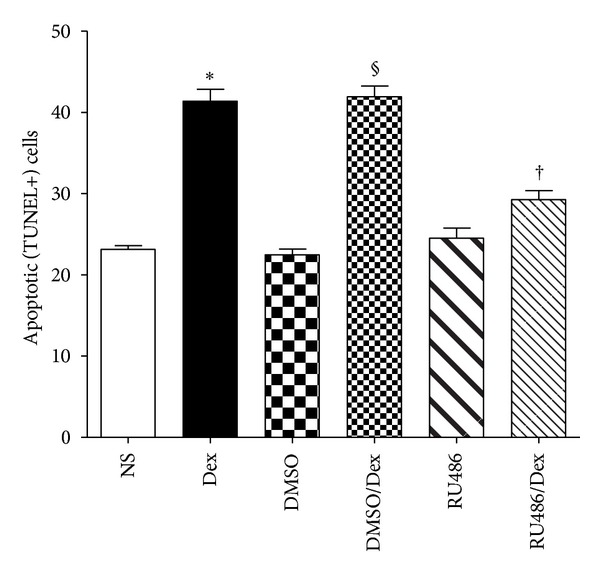
Dexamethasone-(Dex-) induced apoptosis in P1 D1D2 pups was reduced by preadministration of RU486. Dex treatment increased apoptotic cell counts in the hippocampus compared to control (*n* = 6, *P < 0.05); P1 D1D2 pups treated with DMSO or DMSO plus Dex showed results similar to those from pups treated with either normal saline (NS) or Dex alone (*n* = 6, ^§^ *P* < 0.05). RU486 treatment showed apoptotic cell counts similar to those from pups treated with NS or DMSO. Pups treated with RU486 plus Dex had lower apoptotic cell counts than those with Dex or DMSO plus Dex (*n* = 6, ^†^ *P* < 0.05). P1 D1D2: P1 pups receiving treatment on postnatal day 1 and sacrificed on day 2.

Western blot analysis showed that the nuclear fractions of GCRs in the hippocampus were upregulated in the Dex-treated D1D2 group (Figure 4). The Dex-retarded developmental gain in body weight was blocked by RU486 while neither Dex nor RU486 affected the brain weight in this group (Table 2). Furthermore, Dex-induced apoptosis in the P1 D1D2 group was reduced by the preadministration of RU486 (Figure 5). Dex treatment alone increased the apoptotic cell count (41.3 ± 0.60) compared to the control (23.1 ± 0.18); *P* < 0.05); the number of apoptotic cells in pups treated with DMSO (22.4 ± 0.3) or DMSO plus Dex (41.9 ± 0.56) was similar to that in pups treated with NS or Dex alone (^§^ *P* < 0.05). RU486 treatment had no additional effect on apoptosis when compared with the NS or DMSO group. Pretreatment with RU486 followed by Dex reduced the apoptotic cell count (29.2 ± 0.45) (*P* < 0.05) (Figure 5).

### 3.5. Identification of Inflammatory Cells

**Figure 6:**
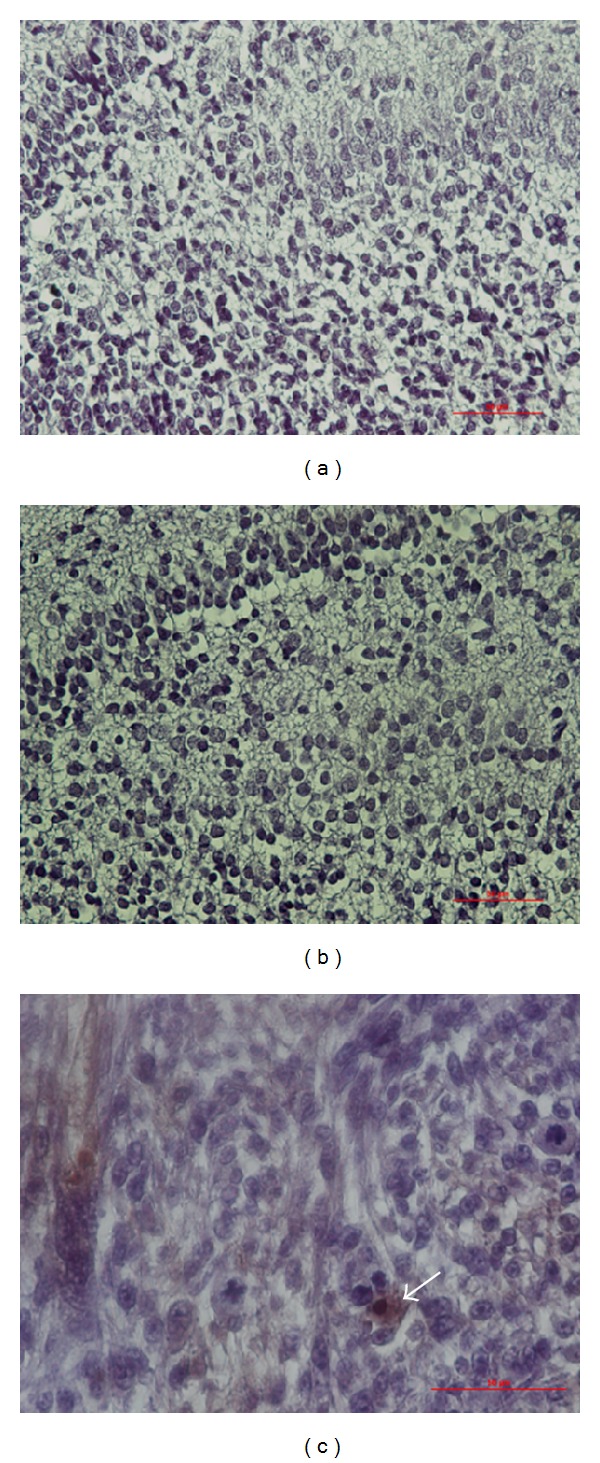
OX-6 IHC staining in the DG. No OX-6 positive cells were identified in P1 D1D2 pups treated with either NS (a) or Dex (b). Brain tumor implant staining as a positive control showed OX-6 positive cells stained brown (c), arrow). Magnification, 400x; scale bar, 50 *μ*m.

Eosin/hematoxylin or IHC staining did not reveal any inflammatory cells in the NS and Dex-treated D1D2. Positive controls after implantation of rat brain tumor cells revealed OX-6 positive cells (stained brown) which helped identification of microglia in the brain (Figure 6).

## 4. Discussion

Steroids have long been used in the treatment of respiratory problems in preterm infants [21]. The complications of adverse neurological effects (such as increased risk of cerebral palsy and neurodevelopment impairment [4, 22, 23] demand the reevaluation of steroid-based therapeutic strategies in postnatal practice [23–26]. The safe timing and dosage of Dex remain undecided for preterm patients [4]. Our results from the P1 rat pups, equivalent to 24-week preterm infants, showed that Dex retarded the developmental gain in body weight, consistent with earlier reports [27–29]. Furthermore, neonatal Dex exposure leads to delayed neurodevelopment and physical maturation, suggesting that Dex permanently alters neuronal functions during this period, particularly those associated with the hypothalamic-pituitary-adrenal axis [27–29]. Other studies showed that neonatal Dex exposure reduces brain weight [10, 28–32]. On the contrary, our data showed no such change. We attribute this discrepancy to the differences in timing, dosage regimes, and/or the preparation of Dex [28, 31, 32]. Among clinicians, the general consensus is that a lower dose of Dex (0.1–0.2 mg/kg/day) facilitates tracheal extubation and reduces the risk of chronic-lung disease. This study revealed no deleterious effects on the brain with single low doses of Dex (0.1–0.2 mg/kg), although it became harmful at a higher dose (0.5 mg/kg). The subgranular zone of the DG contains a reserve of neuroglial progenitor cells [33]. Apoptosis is crucial during neuronal development by eliminating excess cells and ensuring proper synaptic connectivity [34, 35]. GCRs are known to be involved in Dex-induced apoptosis [36] and are present at high levels in the hippocampus where progenitor cells capable of dividing reside in the DG. Our results showed increased apoptosis throughout the DG, suggesting that perinatal development in the hippocampus is vulnerable even to a single dose of Dex when given at a critical time. How this effect is related to the GCR density, receptor types, and the proliferation of progenitor cells remains to be studied.

It has been reported that administration of 3.0 mg/kg Dex to P7 mice increases the apoptosis of cerebellar progenitor cells and reduces the number of cerebellar neurons [37, 38]. It is likely that the dosage and timing of Dex treatment and vulnerability of neural progenitor cells to glucocorticoids together determine the effects on neonatal brain development.

Exposure to Dex in the neonatal period results in marked apoptosis among the nestin-expressing cells in the DG [39]. The importance of Dex-induced apoptosis in the hippocampus and the type of cells affected remain unknown. Our results from double staining in the Dex-treated pups showed higher ratios of cells coexpressing TUNEL and nestin to TUNEL-positive cells, indicating that Dex-induced apoptosis affects the neuroprogenitor cells. This result is consistent with previous reports that neuroprogenitor cells are sensitive to Dex during the early neonatal period [39, 40]. Since the ratio of TUNEL and NeuN coexpressing cells to TUNEL-positive cells also increased, Dex treatment might also cause apoptosis in mature neurons and be associated with a transient and acute slowing of cell proliferation during hippocampal development [41, 42]. Whether these neurons were derived from the proliferating progenitors cells or represent existing mature neurons in the DG remains to be determined.

Mifepristone (or RU-486) is a synthetic steroid with both antiprogesterone and antiglucocorticoid properties. In a recent study, mifepristone was the only GCR antagonist found to increase both mineralocorticoid receptor (MCR) and GCR binding in the rat frontal cortex [43]. The effects of mifepristone on corticosteroid receptor expression could explain the neurocognitive improvement it is reported to induce [43, 44]. RU486 has no effect on Dex regarding thymocyte composition and maturation [45]. MCR-mediated responses to glucocorticoids, rather than GCRs, are important in steroid-responsive hearing disorders [46]. GCR activity levels are high in the hippocampus during the first postnatal week [40]. The anti-inflammatory effects of glucocorticoids require the presence of GCRs [47]. Interestingly, results of these studies and those of our own (60% reduction in Dex-induced apoptosis by RU486) are consistent, suggesting that the type of receptor and the timing of Dex treatment determine the effects of glucocorticoids on the hippocampus. Examining pup brain tissues stained with eosin/hematoxylin or IHC revealed no inflammatory cells in the NS and Dex-treated D1D2 groups. These results suggest that while GCRs are likely to be key players in Dex-induced neuroprogenitor cell death, inflammatory cells are not.

In summary, brain development is a dynamic process in which the growth spurt, differentiation, and cell responses to endogenous (and exogenous) steroids occur at critical times. Species differences add further complexity to the process. These species-specific developmental schedules allowed the design of animal models to study the effects of drug treatment in preterm babies, as in this study. Here, we have demonstrated that timing is a major factor in determining Dex-induced apoptosis in the hippocampus, the vulnerable cells are neuronal precursors, and the process is partly regulated by GCRs. We also provided cytological evidence that the administration of a single dose of Dex can result in deleterious effects in the brain.

## Figures and Tables

**Table 1:** Effects of dexamethasone on rat pup body weight and brain weight.

Age (D)	Body weight, Mean ± SE, g (*N* = 6)		Brain weight, Mean ± SE, g (*N* = 6)	
	NS	Dex	NS	Dex
P1 group				
1^a^	7.17 ± 0.07	6.58 ± 0.06		
2	8.13 ± 0.04	6.63 ± 0.03	0.373 ± 0.013	0.376 ± 0.008
3	9.33 ± 0.14	7.50 ± 0.07*	0.387 ± 0.008	0.373 ± 0.006
5	12.45 ± 0.11	9.83 ± 0.16^†^	0.530 ± 0.013	0.549 ± 0.011
7	16.90 ± 0.33	12.70 ± 0.23^†^	0.699 ± 0.017	0.721 ± 0.007

*N*: number of pups; ^a^days of normal saline (NS) or dexamethasone (Dex) administration to the rat pups.

In P1 group, after Dex or NS injection on day 1, the pups were sacrificed on days 2, 3, 5, and 7.

Compared pups injected with dexamethasone (Dex) and normal saline (NS) by using two-way ANOVA.

*P < 0.05; ^†^ *P* < 0.001.

**Table 2:** Effects of dexamethasone and RU486 on D1D2 rat pup body weight and brain weight.

Treatment	Body weight, Mean ± SE, g (*N* = 6)	Brain weight, Mean ± SE, g, (*N* = 6)
NS	7.75 ± 0.04	0.314 ± 0.004
Dex	6.75 ± 0.08*	0.293 ± 0.004
DMSO	7.50 ± 0.09	0.309 ± 0.004
RU486	7.16 ± 0.08	0.312 ± 0.004
DMSO/Dex	6.83 ± 0.04	0.301 ± 0.004
RU486/Dex	7.08 ± 0.03	0.319 ± 0.004

*N*: number of pups.

Compared pups injected with dexamethasone (Dex) and normal saline (NS) by using Wilcoxon rank sum test, *P < 0.05.

D1D2: administration on postnatal day 1 and sacrificed on day 2; RU486: mifepristone;

DMSO: dimethyl sulfoxide; DMSO/Dex: dimethyl sulfoxide plus dexamethasone;

RU486/Dex: mifepristone plus dexamethasone.

## List of Abbreviations

Dex: Dexamethasone
GCR: Glucocorticoid receptor
DG: Dentate gyrus
CA: Cornu ammonias
RU486: Mifepristone
BDNF: Brain-derived neurotrophic factor
NS: Normal saline
P1/P0: Day1/day 0 newborn pups
D1D2: Pups receiving dexamethasone injection on day 1 and sacrificed on day 2
IHC: Immunohistochemical
IF: Immunofluorescence
TUNEL: Terminal deoxynucleotidyl transferase dUTP nick end labeling.
